# Strategies for Overcoming Bacterial Resistance to Nanoparticles: A Systematic Review

**DOI:** 10.7759/cureus.78064

**Published:** 2025-01-27

**Authors:** Pedro Rosales García, Eva Concepción Aguirre López, Marco Antonio Reyes Torres, Miguel Ángel Noyola Frías, Adriana Torre Delgadillo, Alan Martínez Zumarán, Nuria Patiño-Marín, Marco Felipe Salas Orozco

**Affiliations:** 1 Dentistry, Faculty of Stomatology, Northeastern Regional Complex, Meritorious Autonomous University of Puebla, Puebla, MEX; 2 Dentistry, Clinical Research Laboratory, Faculty of Stomatology, Autonomous University of San Luis Potosí, San Luis Potosí, MEX; 3 Dentistry, Universidad Cuauhtémoc San Luis Potosí, San Luis Potosí, MEX; 4 Oral and Maxillofacial Surgery, Faculty of Stomatology, Autonomous University of San Luis Potosí, San Luis Potosí, MEX; 5 Orthodontics and Dentomaxillofacial Orthopedics, Faculty of Stomatology, Autonomous University of San Luis Potosí, San Luis Potosí, MEX; 6 Dentistry, Clinical Research Laboratory, Faculty of Stomatology, Autonomous University of San Luis Potosi, San Luis Potosí, MEX; 7 Dentistry, Autonomous University of San Luis Potosi, San Luis Potosí, MEX

**Keywords:** antimicrobial strategies, bacterial resistance, functionalized nanoparticles, nanoparticles, systematic review

## Abstract

The increasing prevalence of bacterial resistance to conventional antimicrobial agents and nanoparticles (NPs) has become a critical global health challenge. This systematic review aimed to evaluate strategies for overcoming bacterial resistance to nanoparticles by synthesizing evidence on nanoparticle composition, functionalization, and targeted resistance mechanisms. A comprehensive literature search of studies published between 2000 and 2025 was conducted, focusing on experimental designs assessing antimicrobial efficacy, biofilm disruption, and efflux pump inhibition. The reviewed studies highlighted advanced nanoparticle formulations, including silver-cyanographene conjugates, lanthanum-functionalized graphene oxide, and carbon nanogels, which demonstrated enhanced efficacy against resistant bacterial strains. Key findings emphasized the role of surface functionalization, nanoparticle size modulation, and combination therapies in mitigating resistance. Functionalized nanoparticles effectively disrupted biofilm matrices, inhibited efflux pumps, and enhanced intracellular penetration, reducing bacterial survival rates. Despite promising results, challenges remain, including variability in nanoparticle formulations, limited scalability, and long-term ecological impacts. This review underscores the potential of innovative nanoparticle designs and combination therapies to combat bacterial resistance and emphasizes the need for standardized protocols and clinical translation.

## Introduction and background

The increasing prevalence of bacterial resistance to conventional antimicrobial agents has become a critical global health challenge. According to the World Health Organization (WHO), antimicrobial resistance (AMR) is a leading threat to public health, with predictions estimating that, by 2050, AMR-related deaths could surpass those from cancer if current trends persist. This alarming scenario necessitates the urgent development of innovative approaches to combat resistant bacterial strains [1]. Among the most promising strategies is the application of nanoparticles (NPs) in antimicrobial therapies, leveraging their unique physicochemical properties, such as high surface area-to-volume ratio, functional versatility, and targeted delivery capabilities [2].

Nanoparticles, particularly metallic and polymeric types, have demonstrated significant antimicrobial efficacy through diverse mechanisms, including direct bacterial membrane disruption, generation of reactive oxygen species (ROS), and enhanced drug delivery. However, the increasing reliance on NPs in therapeutic applications has led to emerging evidence of bacterial resistance against these nanomaterials. This resistance is influenced by several factors including bacterial adaptability, biofilm formation, and efflux pump activation. Furthermore, the heterogeneity in nanoparticle formulations and inconsistent protocols in their application contribute to the complexity of understanding and mitigating bacterial resistance [3].

Despite the advances in nanoparticle technology, several challenges remain. First, the variability in nanoparticle formulations, including size, shape, and surface properties, complicates the standardization of their application and the interpretation of research findings. Second, the potential for long-term ecological and health impacts, including the induction of secondary resistance mechanisms, remains poorly understood. Third, the cost and scalability of nanoparticle production and functionalization limit their widespread application, particularly in resource-constrained settings. These gaps highlight the need for a systematic evaluation of current strategies to overcome bacterial resistance to nanoparticles, providing a framework for future research and clinical translation [4]. This systematic review addresses these critical gaps by synthesizing existing evidence on strategies to overcome bacterial resistance to nanoparticles.

## Review

This systematic review was conducted following the Preferred Reporting Items for Systematic Reviews and Meta-Analyses (PRISMA) guidelines [5].

Eligibility criteria

Studies meeting the eligibility criteria were included, with the criteria defined using the PICOS framework as follows: (1) Population - studies investigating bacterial strains exhibiting resistance to antimicrobial nanoparticles, including both planktonic and biofilm-associated bacteria. (2) Intervention - strategies employed to overcome bacterial resistance to nanoparticles, such as surface functionalization, combination therapies, or synergistic agents. (3) Comparators - studies with control groups utilizing non-functionalized nanoparticles or conventional antimicrobials. (4) Outcomes - efficacy in reducing bacterial resistance, measured through minimum inhibitory concentration (MIC) reductions, biofilm disruption, or bactericidal activity. (5) Study design - experimental studies (in vitro or in vivo) and clinical studies. Only articles published in the English language between 2000 and 2025 were included to ensure the analysis was focused on contemporary advancements. Non-peer-reviewed articles, editorials, and conference abstracts were excluded.

Search strategy

A comprehensive electronic search was conducted across the following databases: PubMed, Scopus, and Web of Science, supplemented by manual searches of reference lists in included studies. The search strategy employed a combination of keywords and Boolean operators. For PubMed, the keywords were as follows: (bacterial resistance OR antimicrobial resistance OR biofilm resistance) AND (nanoparticles OR nanomaterials OR nanotechnology) AND (strategies OR methods OR approaches OR techniques). For Scopus and Web of Science, the keywords were as follows: ("bacterial resistance" OR "antimicrobial resistance") AND "nanoparticles" AND "mechanisms". The final search was performed in January 2025.

Study selection

Two independent reviewers screened the titles and abstracts of all retrieved records for relevance. Studies meeting the inclusion criteria underwent full-text review. Discrepancies during this process were resolved through discussion, and a third reviewer acted as an arbitrator when necessary.

Data extraction

Data were extracted using a standardized form that was pre-tested on a subset of studies to ensure consistency and accuracy. The extracted information included study characteristics such as authors, year of publication, and study design; details about the nanoparticles, including their composition, size, and functionalization; the resistance mechanisms targeted, such as biofilm disruption, efflux pump inhibition, or other strategies; and outcomes, including MIC reduction, biofilm inhibition percentages, and bactericidal effects. Two reviewers independently conducted the data extraction process, and any discrepancies were resolved through consensus or, when necessary, consultation with a third reviewer.

Data analysis

Due to the heterogeneity of the included studies, a meta-analysis was not feasible. The studies varied significantly in terms of their experimental designs, bacterial strains tested, methods used to evaluate resistance mechanisms, and outcome measures. As a result, the data were synthesized narratively, focusing on identifying common patterns and trends across the studies. Key findings from each study are presented in tabular form for clarity and comparison.

Risk of bias assessment

The risk of bias in the included studies was assessed using the Risk of Bias 2 (RoB 2) tool, a validated instrument. RoB 2 assesses bias across five key domains related to study design, conduct, and reporting. Each domain was evaluated systematically as described further. The first domain, bias arising from the randomization process, examined whether the random sequence generation and allocation concealment were adequately performed to minimize selection bias. The second domain, bias due to deviations from intended interventions, assessed whether the interventions were implemented as planned and whether deviations were likely to impact outcomes systematically. The third domain, bias due to missing outcome data, focused on whether the amount and handling of missing data could lead to biased results. The fourth domain, bias in the measurement of the outcome, evaluated whether the measurement methods used were reliable and applied equally across study groups. The fifth and final domain, bias in the selection of the reported result, assessed whether all prespecified outcomes were reported and whether the results reported were selective.

For each domain, a series of signaling questions were answered, and responses were categorized as “low risk of bias,” “some concerns,” or “high risk of bias.” Each domain's judgment was made independently by two reviewers with expertise in systematic reviews and clinical trial evaluation. Any discrepancies in judgments were resolved through discussion or, when necessary, by consulting a third reviewer. The overall risk of bias for each study was determined according to the RoB 2 guidelines. A study was considered to have a “low risk of bias” if all domains were judged as low risk. It was categorized as having “some concerns” if at least one domain raised concerns, but no domain was judged as high risk. A study was classified as having a “high risk of bias” if one or more domains were rated as high risk. The results of the risk of bias assessment were documented in a structured table that included the ratings for each domain and the overall risk of bias for each study.

Results

**Figure 1:**
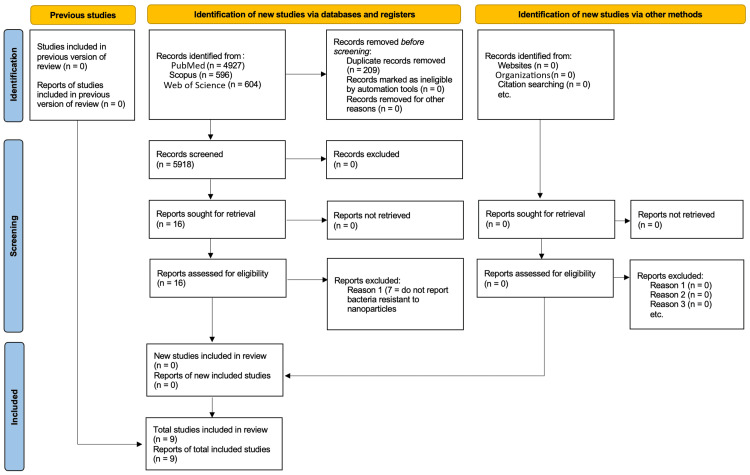
PRISMA flow diagram of study selection process. PRISMA: Preferred Reporting Items for Systematic Reviews and Meta-Analyses

The studies summarized in Table 1 demonstrate a range of innovative strategies to overcome bacterial resistance to nanoparticles (NPs). These approaches include functionalized nanoparticles, such as chitosan, silver, graphene oxide, and carbon nanogels, targeting mechanisms like biofilm disruption, efflux pump inhibition, and membrane damage. Efflux pump inhibitors and agents like Triton X-100 enhanced the efficacy of silver ions, while functionalized materials, such as graphene oxide with lanthanum or cyanographene-bound silver, showed improved antibacterial potency and reduced resistance development. Nanoparticles with targeted physicochemical properties, such as size modulation (4,6-diamino-2-pyrimidinethiol-capped gold nanoparticles {AuDAPTs} and carbon nanogels {CNGs}), disrupted bacterial membranes and reduced resistance evolution. Across the studies, these strategies were effective in enhancing bacterial susceptibility, maintaining nanoparticle activity over multiple bacterial generations, and minimizing cytotoxicity, highlighting their potential for combating multidrug-resistant pathogens. Figure 1 presents the PRISMA flow diagram illustrating the systematic selection process.

**Table 1:** Experimental studies on strategies to overcome bacterial resistance to nanoparticles.

Studies	Year	Study design	Nanoparticle composition	Nanoparticle size	Functionalization	Resistance mechanisms targeted	Outcomes
Upadya et al. [6]	2011	Experimental study evaluating the role of efflux pump inhibitors (EPI) in enhancing antimicrobial efficacy	Chitosan nanoparticles (CNPs)	Not applicable	Used in combination with calcium hydroxide and light-activated disinfection (LAD)	Efflux pump inhibition, biofilm matrix disruption	EPI enhanced the antibiofilm effect of LAD and low concentrations of Ca(OH)₂; LAD at 40 J/cm² completely inactivated Enterococcus faecalis biofilms; EPI had no impact on high Ca(OH)₂ or CNPs
Panáček et al. [7]	2017	Experimental study on bacterial resistance mechanisms to silver nanoparticles (AgNPs)	Silver nanoparticles (AgNPs)	~28 nm	Synthesized via Tollens process; unmodified	Flagellin-induced aggregation, biofilm matrix alteration	Development of bacterial resistance via aggregation induced by flagellin; resistance suppressed using pomegranate rind extract to inhibit flagellin production
Zheng et al. [8]	2019	Experimental study evaluating engineered graphene oxide nanocomposites (La@GO) against antimicrobial resistance	Graphene oxide with lanthanum hydroxide (La@GO)	12.1-245.2 nm	Functionalized with lanthanum hydroxide nanoparticles (La{OH}₃)	Biofilm disruption, lipid peroxidation, phospholipid dephosphorylation, and peptidoglycan disruption	Potent antibacterial effects without inducing resistance after 30 days of sub-MIC exposure; reduced biofilm and bacterial viability
Cui et al. [9]	2020	Experimental study on silver resistance in E. faecalis and the role of simvastatin in overcoming it	Silver ions and silver nanoparticles (Ag+/AgNPs)	<5 nm	Not specified; tested with simvastatin as an adjunct	EPS-mediated silver resistance, biofilm protection, intracellular silver ion penetration	Simvastatin reduced EPS-mediated silver resistance, allowing intracellular penetration of Ag+/AgNPs and enhanced bactericidal effects against resistant E. faecalis
Zheng et al. [10]	2021	Experimental study on bacterial resistance to nanoparticles	Gold nanoparticles (AuDAPTs)	1.8-26.9 nm	Capped with 4,6-diamino-2-pyrimidine thiol (DAPT)	Biofilm disruption, membrane damage, and surface structure alteration	MIC reduction with up to 16-fold resistance induction for AuDAPTs; improved biofilm inhibition and bacterial damage via size modulation
Panáček et al. [11]	2021	Experimental study evaluating silver covalently bound to cyanographene against multidrug-resistant bacteria	Silver nanoparticles bound to cyanographene (GCN/Ag)	4-8 nm	Functionalized with nitrile groups on cyanographene surface	Bypassing flagellin-induced aggregation, biofilm disruption, and membrane interaction	Thirty-fold higher antibacterial potency against AgNP-resistant bacteria; maintained activity over 60 bacterial generations; and high cytocompatibility and low silver leaching
Mao et al. [12]	2022	Experimental study using carbon nanogels (CNGs) against resistant bacteria	Carbon nanogels (CNGs) derived from L-lysine hydrochloride	120±22.3 nm (CNGs-270)	Nitrogen and chlorine doping, positively charged structures	Membrane disruption, oxidative stress elevation, suppression of resistance evolution	CNGs-270 exhibited superior antibacterial activity compared to Ag NPs and antibiotics, with negligible resistance development after 20 bacterial passages
Lv et al. [13]	2024	Experimental study on the effect of Triton X-100 combined with Ag+ against resistant E. faecalis	Silver ions (Ag+) combined with Triton X-100	Not applicable	Membrane permeability enhancement using Triton X-100	Efflux pump suppression, membrane integrity disruption	The combination of TX-100 and Ag+ significantly reduced resistance by enhancing Ag+ entry into cells and disrupting bacterial membranes
Sun et al. [14]	2025	Experimental study on nanosilver resistance mechanisms	Silver nanoparticles (AgNPs)	20.97 nm	Targeted physicochemical interventions	Flagellin-mediated precipitation, copper efflux pump activation	Physicochemical interventions reduced resistance by up to 10,000-fold and prevented resistance evolution

Table 2 summarizes the risk of bias assessment for multiple studies evaluated using the RoB 2 tool. Each study was analyzed across five key domains as follows: bias arising from the randomization process, bias due to deviations from intended interventions, bias due to missing outcome data, bias in the measurement of the outcome, and bias in the selection of the reported result. The overall risk of bias for each study was also determined. The majority of studies demonstrated a low risk of bias across most domains, indicating a generally robust methodological quality. However, "some concerns" were noted in the domains of bias due to deviations from intended interventions and bias in the measurement of outcomes, which highlights potential areas for methodological improvement. Despite these concerns, the overall risk of bias was rated as low for all studies, suggesting that the findings are reliable and minimally affected by bias.

**Table 2:** Summary of risk of bias assessment across studies using the RoB 2 tool. RoB 2 tool: Risk of Bias 2 tool

Studies	Bias arising from the randomization process	Bias due to deviations from intended interventions	Bias due to missing outcome data	Bias in measurement of the outcome	Bias in selection of the reported result	Overall risk of bias
Upadya et al. [6]	Unclear	Low	Low	Some concerns	Low	Low
Panáček et al. [7]	Low	Some concerns	Low	Some concerns	Low	Low
Zheng et al. [8]	Low	Some concerns	Low	Some concerns	Low	Low
Cui et al. [9]	Low	Some concerns	Low	Some concerns	Low	Low
Zheng et al. [10]	Low	Some concerns	Low	Some concerns	Low	Low
Panáček et al. [11]	Low	Some concerns	Low	Some concerns	Low	Low
Mao et al. [12]	Low	Some concerns	Low	Some concerns	Low	Low
Lv et al. [13]	Low	Some concerns	Low	Some concerns	Low	Low
Sun et al. [14]	Low	Some concerns	Low	Some concerns	Low	Low

Discussion

Flagellin serves as a critical structural and functional protein in bacteria, forming the main component of the flagellar filament, which is essential for motility. This motility allows bacteria to navigate their environment, move toward favorable conditions (chemotaxis), and evade host immune responses [15]. Beyond locomotion, flagellin plays a vital role in bacterial adhesion and biofilm formation, enhancing the ability of bacteria to colonize surfaces and establish infections [16,17]. In addition to its structural role, flagellin is a potent immunostimulatory molecule recognized by the host immune system, specifically through toll-like receptor 5 (TLR5). This recognition triggers innate immune responses, including the production of pro-inflammatory cytokines, which help the host identify and combat bacterial infections. Interestingly, some bacteria modulate flagellin expression to evade immune detection, underscoring its dual role in both virulence and immune interaction. Thus, flagellin is integral to bacterial survival, adaptability, and pathogenicity [18]. The study by Panáček et al. demonstrated that bacterial resistance to AgNPs can arise through phenotypic changes, particularly the production of flagellin, an adhesive protein. The mechanism involves extracellular secretion of flagellin, which adsorbs onto the surfaces of AgNPs, triggering their aggregation and sedimentation. This results in reduced antibacterial activity as aggregated nanoparticles lose their ability to interact with bacterial membranes. Unlike ionic silver, which retains antibacterial efficacy, AgNP aggregation appears to be specific to nanoparticulate forms of silver​. Despite attempts to stabilize AgNPs using surfactants and polymers, resistant strains of *Escherichia coli* and *Pseudomonas aeruginosa *exhibited no significant reduction in resistance, highlighting the robustness of the flagellin-mediated mechanism [7]. To counteract this resistance, inhibitors of flagellin production have been identified as promising strategies. The use of pomegranate rind extract (PGRE), a natural flagellin inhibitor, successfully restored the antibacterial activity of AgNPs by suppressing flagellin synthesis [19]. When combined with AgNPs, PGRE reduced the minimal inhibitory concentrations (MICs) of resistant strains to levels comparable to those of susceptible strains (MIC for *E. coli* CCM 3954: 3.38 mg/L vs. resistant MIC: 108 mg/L without PGRE) [7]. Flagellin inhibitors, such as sub-inhibitory polymyxin B (PmB), have been used to reduce biofilm formation, a key bacterial resistance mechanism. For example, in Vibrio cholerae, PmB disrupted flagellar function by decreasing the number of flagella on the bacterial surface, leading to reduced motility and impaired attachment, both essential for biofilm initiation. Additionally, PmB caused flagellin to accumulate in the secretome, further compromising flagellar assembly. By targeting flagellin and preventing surface adhesion, these inhibitors effectively halted the early stages of biofilm formation, thereby counteracting two critical mechanisms of bacterial resistance [20]. Future work should focus on optimizing nanoparticle formulations and identifying other natural or synthetic inhibitors of bacterial aggregation processes to ensure the long-term efficacy of nanoparticle-based antimicrobial strategies.

Unlike conventional antibiotics and silver nanoparticles, which promote resistance evolution, La@GO nanocomposites demonstrated consistent bactericidal efficacy over long-term sub-MIC exposure, with no detectable genetic mutations or resistance acquisition in *E. coli *(AMR). The synergy between La(OH)_3_ and graphene oxide enhances these effects, making La@GO a robust antimicrobial agent. For example, after 30 days of exposure, *E. coli *strains exposed to La@GO maintained their susceptibility, while those treated with silver nanoparticles or antibiotics like norfloxacin showed significant increases in MIC values, reflecting acquired resistance [8]. Graphene oxide nanocomposites (La@GO) present a promising solution due to their unique extracellular multitarget invasion (EMTI) mechanism, effectively countering resistant bacterial strains without inducing secondary resistance. This mechanism involves lipid peroxidation, phospholipid dephosphorylation, and peptidoglycan disruption, targeting the bacterial membrane and cell wall instead of relying on intracellular pathways​ [21].

Simvastatin, primarily known for its cholesterol-lowering properties, disrupts the extracellular polymeric substance (EPS) barrier, allowing Ag+ and AgNPs to penetrate bacterial cells effectively. Electron microscopy and element mapping revealed that in the presence of simvastatin, silver particles no longer aggregated in the EPS but entered the bacterial cells, significantly enhancing bactericidal activity. Additionally, Cui et al. demonstrated in their study that resistance mechanisms between Ag+ and AgNPs are interrelated. Silver-resistant strains exhibited higher minimum bactericidal concentrations (MBCs) for both Ag+ and AgNPs, suggesting shared pathways of resistance. This underscores the necessity of targeting the EPS barrier for both nanoparticle and ion-based antimicrobial strategies. Simvastatin's synergistic effects were quantified through reductions in MBCs and colony-forming units (CFUs). For silver-resistant strains, simvastatin decreased the MBC of Ag+ from 600 mg/L to 200 mg/L and that of AgNPs from 130 mg/L to 40 mg/L. These findings confirm that simvastatin not only enhances silver's bactericidal properties but also mitigates resistance development​ [9].

The bactericidal effects of simvastatin have been previously studied, highlighting its potential as an antimicrobial agent. Simvastatin disrupts bacterial macromolecular synthesis, including DNA, RNA, protein, and lipid production, by interfering with key enzymes and regulatory pathways. Proteomic studies show it induces protein degradation, disrupting bacterial energy metabolism and homeostasis while sparing mammalian cells. Simvastatin also reduces bacterial virulence by suppressing toxin production in *Staphylococcus aureus*, such as α-hemolysin, which is essential for tissue damage. Additionally, it exhibits strong antibiofilm activity by inhibiting bacterial adhesion and extracellular polysaccharide production, both critical for biofilm formation and resistance. It also degrades existing biofilms, enhancing the efficacy of other antimicrobial agents. While primarily effective against Gram-positive bacteria like MRSA, simvastatin shows improved activity against Gram-negative bacteria when combined with agents like colistin. These findings, combined with its ability to reduce inflammation and enhance treatment efficacy, highlight simvastatin's versatility as an antibacterial agent for addressing biofilm-associated and multidrug-resistant infections [22].

The study by Zheng et al. showed that modulating the size of DAPT-capped gold nanoparticles (AuDAPTs) slows bacterial resistance compared to antibiotics like gentamicin. Resistance involved structural changes in bacterial surfaces, not genetic mutations, and was size-specific. Smaller AuDAPTs (e.g., 1.8 nm) were more effective, showing minimal MIC increases compared to larger particles. In wound dressings, smaller AuDAPTs reduced bacterial loads and improved healing, highlighting size adjustment as a practical strategy for combating drug-resistant infections [10]. Previous studies have shown that nanoparticle size greatly influences their antimicrobial efficacy, with smaller nanoparticles being more effective due to their increased surface area and membrane interaction. In the study by Kumari et al., biogenic silver nanoparticles (AgNPs) synthesized using *Trichoderma viride* confirmed this, showing size- and shape-dependent activity against multidrug-resistant (MDR) pathogens like *Escherichia coli *and *Staphylococcus aureus*. Spherical AgNPs (2-5 nm) were the most effective, disrupting membranes, inhibiting proteins, and generating reactive oxygen species (ROS), while larger particles (50-100 nm) were less potent. Smaller AgNPs also synergized with antibiotics like ampicillin, enhancing their effectiveness against resistant strains [23]. In the study by Huang et al., selenium nanoparticles (Se NPs) demonstrated size-dependent antimicrobial efficacy, with 81 nm particles showing the highest activity against methicillin-sensitive and methicillin-resistant *Staphylococcus aureus *(MSSA and MRSA) [24].

In the study by Panáček et al., silver covalently bound to cyanographene (GCN/Ag) was shown to overcome bacterial resistance to silver nanoparticles (AgNPs). Unlike conventional AgNPs, GCN/Ag bypasses resistance mechanisms such as flagellin-mediated aggregation by immobilizing silver ions on cyanographene, preventing leaching and ensuring stable interaction with bacterial membranes. This interaction disrupts membrane integrity, induces reactive oxygen species (ROS) production, and leads to cell death without deep penetration into bacterial cells. GCN/Ag exhibited significantly enhanced efficacy, with minimum inhibitory concentrations (MICs) up to 30-fold lower than AgNPs for multidrug-resistant *Escherichia coli *and *Pseudomonas aeruginosa*. Moreover, GCN/Ag maintained antibacterial activity over 60 bacterial generations, unlike AgNPs, which lost efficacy after 20 generations. Its high cytocompatibility, achieved through minimized silver ion leaching, reduces cytotoxicity to human cells, making GCN/Ag a safer and sustainable option for antimicrobial applications [11]. Cyanographene has also been shown to enhance antibiotic efficacy against resistant bacteria. In the study by Hochvaldová et al., cyanographene-silver nanohybrids (GCN/Ag) effectively overcame resistance by preventing nanoparticle aggregation and enhancing membrane interaction. Combined with antibiotics like gentamicin and ceftazidime, GCN/Ag reduced MICs up to 32-fold against multidrug-resistant *E. coli *and *P. aeruginosa*. This nanohybrid demonstrated strong synergy with antibiotics, restoring their efficacy with minimal cytotoxicity, highlighting its potential for combating antibiotic resistance [25].

In the study by Mao et al., carbon nanogels (CNGs), synthesized via controlled pyrolysis of L-lysine hydrochloride, were shown to be a novel antimicrobial strategy against drug-resistant bacteria [12]. CNGs exert their effects through multiple mechanisms, including reactive oxygen species (ROS) generation, disruption of membrane potential, and direct physical damage to bacterial membranes. Their positively charged, nitrogen-doped structure interacts with negatively charged bacterial cell walls, destabilizing membrane integrity and causing cell death. Unlike silver nanoparticles and conventional antibiotics, which often face resistance due to genetic adaptations or extracellular polymeric substance (EPS) barriers, CNGs suppress resistance evolution, maintaining stable minimum inhibitory concentrations (MICs) over 20 bacterial passages. They also demonstrate high biocompatibility with mammalian cells and minimal hemolytic activity, making them a safe and scalable option for clinical use. Nanogels, derived from materials like chitosan, hyaluronic acid, and alginate, demonstrate unique properties such as high biocompatibility, biodegradability, and stimuli-responsiveness, enabling efficient penetration into biofilms [26]. These nanogels have been successfully loaded with various agents, including antibiotics, antimicrobial peptides, and nanoparticles, to target biofilms formed by pathogens like *Staphylococcus aureus *and *Escherichia coli *[27,28]. Their mechanisms include disrupting biofilm extracellular polymeric substances (EPS), generating reactive oxygen species (ROS), and enhancing drug delivery to bacteria. Despite their promise, challenges remain, such as limited exploration of stimuli-responsive behaviors and a narrow spectrum of antibiofilm agents. Future research should focus on multistrain biofilms, novel targeting mechanisms, and integrating nanogels into clinical therapies for effective biofilm eradication [29].

In the study by Sun et al., two resistance mechanisms to silver nanoparticles (AgNPs) were identified as follows: flagellin-mediated precipitation (state I) and the Cus copper efflux pump (state II). State I involved flagellin overproduction, leading to AgNP aggregation, while state II used the Cus system to expel Ag+ ions, reducing their antimicrobial effects. The study proposed disrupting the proton motive force (PMF) with carbonyl cyanide m-chlorophenylhydrazone (CCCP) to block flagellin production and using potassium hydroxide (KOH) or L-arginine to inhibit the Cus efflux pump by destabilizing PMF. Additionally, activating the sigma E (σE) stress response through heat shock treatments sensitized bacteria to AgNPs, reducing survival rates significantly. Several efflux pumps have been linked to bacterial resistance against nanoparticles (NPs), particularly silver nanoparticles (AgNPs) [14]. For example, the SilCBA pump, part of the sil operon, expels Ag+ ions, aiding resistance in bacteria like *Salmonella typhimurium*. The CusCFBA system in *E. coli *removes Ag+ and Cu+ ions through a coordinated protein complex, while the CzcABC system confers resistance to Zn, Cd, and Co, and is activated during exposure to quantum dots and CuO NPs. Additionally, the CopA pump, a CPx-type ATPase, expels Cu and Ag ions, and the AcrAB-TolC pump contributes to resistance against Zn and TiO₂ NPs [3]. Efflux pump inhibitors have also been shown to be useful in overcoming resistance to nanoparticles [6].

In the study by Lv et al., Triton X-100 (TX-100), a non-ionic surfactant, was investigated for its ability to enhance the antibacterial activity of silver ions (Ag+) against *Enterococcus faecalis*, including silver-resistant strains (AREf) [13]. TX-100 disrupted bacterial membranes by forming micelles above its critical micelle concentration (CMC), creating pores that increased Ag+ uptake. Transmission electron microscopy confirmed extensive membrane damage, while ICP-MS analysis showed higher intracellular Ag+ levels in the presence of TX-100. This combination significantly lowered the minimum inhibitory and bactericidal concentrations of Ag+, reducing resistance and cytotoxicity associated with high silver doses. TX-100's ability to reverse silver resistance highlights its potential as a safer and effective strategy for combating bacterial resistance, particularly in clinical applications like root canal disinfection [13]. Previous studies have demonstrated that combining Triton X-100 (TX-100) with other antimicrobial agents enhances their bactericidal activity. In the study by He et al., TX-100 improved the efficacy of gentamicin sulfate and ciprofloxacin hydrochloride against *Enterococcus faecalis *by increasing membrane permeability, disrupting membrane potential, and downregulating resistance-related genes like ABC transporters [30]. Similarly, Duan et al. demonstrated that TX-100 combined with silver ions (Ag+) significantly enhanced antibacterial effects against planktonic and biofilm *E. faecalis *with lower cytotoxicity compared to 2% chlorhexidine [31]. Finally, *He et al.* also reported that TX-100 and metformin (Met) effectively inhibited *E. faecalis *biofilms in both normal and high-glucose conditions, showing low cytotoxicity and potential as a root canal disinfectant, particularly for diabetic patients. These studies underscore the potential of TX-100 as an adjuvant to enhance antimicrobial efficacy and reduce resistance [32].

## Conclusions

This systematic review demonstrates the potential of functionalized nanoparticles, such as silver-cyanographene conjugates and carbon nanogels, to overcome resistance to nanoparticles by targeting mechanisms like biofilm disruption and efflux pump inhibition. Overcoming resistance to nanoparticles involves enhancing their efficacy against bacterial adaptations, such as aggregation or expulsion, that reduce their antimicrobial activity. These strategies show high efficacy and biocompatibility, but challenges such as variability in formulations and scalability remain. Standardized protocols and further research are needed to advance their clinical application and combat antimicrobial resistance effectively.
